# The Effect of a Child Model on Breath-Sounds Examination Skills and Satisfaction on Nursing Students

**DOI:** 10.3390/healthcare10071165

**Published:** 2022-06-22

**Authors:** Silpthai Thamruangrit, Sermsri Santati, Jumpee Granger, Dongruethai Buadong, Jatuporn Thongsri

**Affiliations:** 1 Ramathibodi School of Nursing, Faculty of Medicine Ramathibodi Hospital, Mahidol University, Bangkok 10400, Thailand; fahsuay_silp@hotmail.com (S.T.); sermsris@hotmail.com (S.S.); doungruthai.bou@mahidol.ac.th (D.B.); 2 Computer Simulation in Engineering Research Group, College of Advanced Manufacturing Innovation, King Mongkut’s Institute of Technology Ladkrabang, Bangkok 10520, Thailand; jatuporn.th@kmitl.ac.th

**Keywords:** breathing sound, child model, Gagné’s learning theory, auscultation skill, learning media innovation, nursing student, respiratory

## Abstract

One of nursing students’ auscultation critical skills is listening to a child’s breathing sounds. Previously, learning this skill required a SimBaby, which was insufficient, causing nursing students to lack proficiency. Therefore, a CHIld Model (CHIM), an innovation emulating breathing sounds, has been invented based on Gagné’s learning theory to solve this insufficiency. This article reports on the CHIM invention, consisting of hardware, software, and programming, and its effect on nursing students’ breath-sounds examination skills and satisfaction. First, the CHIM was assessed for quality and satisfaction by experts. The results were good in quality and had the highest satisfaction for application in actual use. Second, the CHIM was assessed for auscultation skills and satisfaction among nursing students. Forty-four junior nursing students participated. Next, they were randomly divided into experimental and control groups. Then, both were taught the same about respiratory problems with the SimBaby, except the experimental group had training with the CHIM. After that, both groups’ auscultation skills and satisfaction in the experimental group were examined. Finally, the statistical analysis showed that after the intervention was applied, learning with the CHIM was better than without, with the highest satisfaction level. As intended, the CHIM can help effectively enhance students’ learning and proficiency.

## 1. Introduction

The respiratory system is an essential system of body functions to exchange gas. Therefore, an abnormality from a disease or any cause that affects the respiratory system will deplete oxygen. Without proper treatment, the abnormality can lead to respiratory failure and death. Pediatric respiratory tract infections are the number one cause of morbidity and mortality among children worldwide [1]. Especially in children under five years old, since the respiratory system is not fully developed, it may lead to symptoms such as hypoxia more quickly and severely than in adults. Therefore, a quick assessment of symptoms is necessary for pediatric patients to receive the proper care. Respiratory symptoms assessment methods consist of a history of talking and physical examination such as inspection, palpation, percussion, and auscultation. However, a physical examination of children generally uses looking and listening since they tend to cry when employing other assessment methods. Accordingly, the auscultation skill of breathing sounds by listening is the key to examining the child’s body; therefore, all nursing students must be trained and proficient.

**Figure 1:**
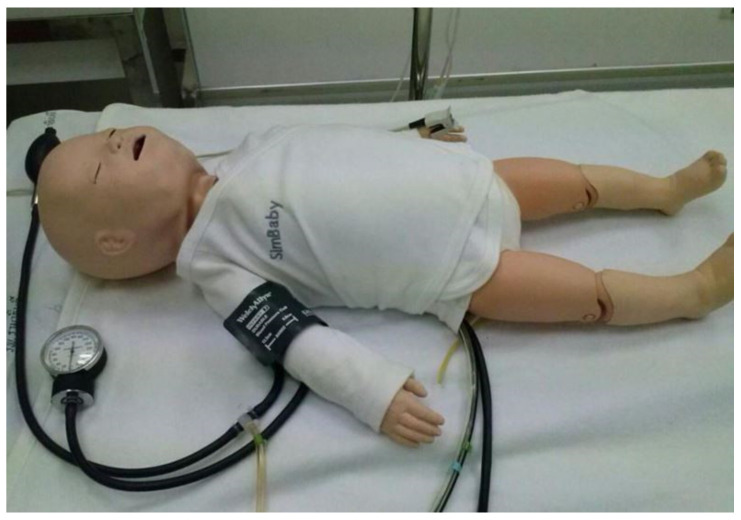
A SimBaby [3].

In leading Thailand nursing schools, high-fidelity simulation (HFS) [2] is employed to train the mentioned skill for all nursing students. A novel healthcare education methodology includes sophisticated life-like mannequins in a realistic patient environment. Therefore, the mannequin is a vital tool to encourage training, leading to a proficient target skill. The “SimBaby [3]” shown in Figure 1 is a mannequin of a Thailand nursing school where the HFS has been employed in training. However, from the authors’ experience of using it for a long time, it has limitations in functions and usability. Most importantly, in terms of usability limitations, the SimBaby must be imported from abroad at an expensive cost. Thus, it is limited in number, not enough for the number of nursing students, which may increase every year. Furthermore, nursing students can strictly practice and review their skills while training only in a laboratory. These limitations cause nursing students not to achieve maximum learning efficiency and are unskillful. Therefore, the lack of mannequins such as the SimBaby is a critical problem that needs to be addressed urgently.

Innovation is a solution to this problem. However, innovation must consist of technological knowledge by engineers and healthcare from nurses, which are of our interest and experience. For example, two authors (S.S. and J.T) have previously developed a modified small-volume jet nebulizer that could help small asthmatic children receive more medication [4]. Computational fluid dynamics in the engineering field was applied to design a novel jet nebulizer, and healthcare in nursing was employed to determine the optimal condition for use in patients. The mentioned research has been recognized for its practicality, encouraging us to try to invent an innovation to address this problem.

Accordingly, the CHIld Model (CHIM), a low-cost training tool for emulating breathing sounds, has been invented. Therefore, this article reports on the concept designs of the CHIM, including hardware, software, and programming, and its effect on breath-sounds examination skills and the satisfaction of experts and nursing students after actual use. Additionally, a research methodology is described step by step from the beginning of the CHIM design to the end with the outcomes after implementing the CHIM with the statistical assessment.

This research highlights that the CHIM is cheaply assembled from simple devices available in the market. Moreover, the CHIM, instructional manual, and a training process were designed based on Gagné’s learning theory, which is widely accepted and used in learning development. Thus, it is easy and reliable to employ in this and further research. Significantly, the CHIM design was accessed, suggested, and commented on by experts in many fields, such as medical doctors, nurses who have experience in anatomy, physiology, and physical examination of children, and engineers who have expertise in modern technology. From the authors’ review, no research invented a model for the aforementioned purposes. Therefore, this article’s reports are reliable and may be thought-provoking to innovate the readers and innovators.

The research methodology consists of the CHIM invention process and breath-sounds examination skills on nursing students, briefly explained as follows.

## 2. CHIM Invention Process

**Figure 2:**
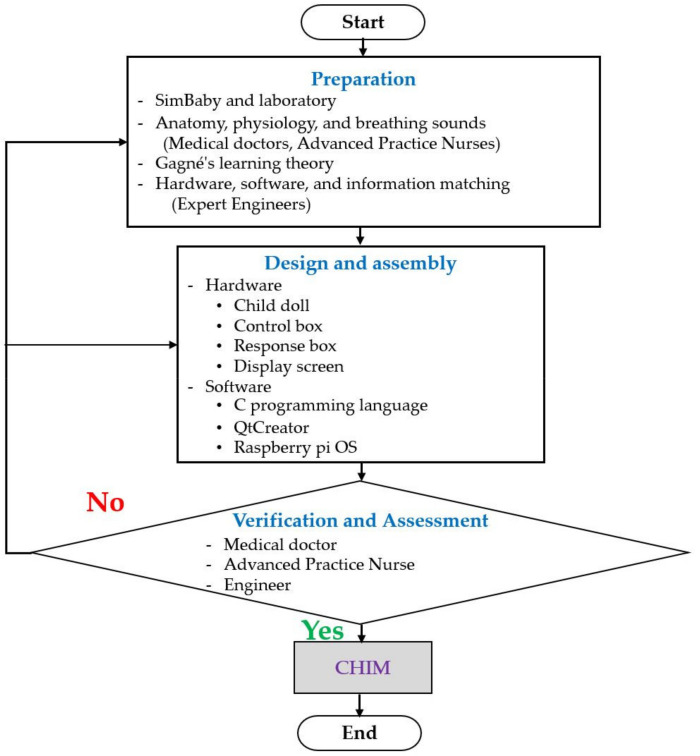
A flowchart of CHIM invention process.

Figure 2 shows the CHIM invention process. There are three phases: preparation, design and assembly, and verification by experts in related fields.

### 2.1. Preparation

Preparation includes a workshop, literature review, and consultation to prepare materials, devices, and engineering knowledge for inventing the CHIM.

#### 2.1.1. SimBaby and Laboratory

To make the CHIM function as close to an actual child’s breathing sounds as possible and achieve effective training, the authors had a workshop with training auscultation skills of listening to breathing sounds using the SimBaby with the same learning and teaching process as nursing students’ laboratory. The workshop results revealed the advantages of the SimBaby, and there are several breathing sounds with a clear voice and adjustable. In addition, its skin is soft and flexible, similar to a natural child, as shown in Figure 1. This skin allows the breathing sounds to spread evenly throughout the SimBaby’s body and easily fit close to the stethoscope. These advantages make it easier for nursing students to practice auscultation skills, but they absolutely make the SimBaby too expensive. On the contrary, the limitation is that the location of breathing sounds has not yet been determined, so nursing students do not know each breathing sound’s accurate position and are confused. Additionally, due to the high price of the SimBaby and the complex electronic control devices inside, nursing students are closely supervised to prevent any damage to the SimBaby. Moreover, nursing students have limited time to review their learning skills and cannot take the SimBaby out of the laboratory. These limitations are the reasons why some nursing students’ learning outcomes are not attainable. Therefore, the CHIM is expected to solve the limitations and help support nursing students’ learning skills and proficiency in child breathing sounds.

In fact, emulating breathing sounds is one of the SimBaby’s capabilities. It also simulates some results of child body functions such as breathing sound, heart sound, lung sound, vocal sounds, brachial pulse, femoral pulse, electrocardiogram (ECG), etc. [3]. These make it expensive and the only one available in Thailand’s top nursing schools. Therefore, in this research, the breathing sounds were focused on.

#### 2.1.2. Anatomy, Physiology, and Breathing Sounds

In order to get rid of the mentioned limitations and help nursing students’ learning, the authors conducted a literature review from textbooks and research articles, suggestions, and comments from consultants such as medical doctors and advanced practice nurses (APNs) who have more experience with the physical examination of children related to the anatomy and physiology, respiratory system, breathing sounds, and type of abnormality. The APN is a nurse who has the expertise and a certificate in a specific field. This research is in the field of nursing patients with the respiratory system. Their essential knowledge is that there are six types of breathing sounds frequently detected in the physical examination of most child patients: normal lung sound, stridor, rhonchi, wheezing, fine crepitation, and coarse crepitation [5,6,7].

Normal lung sound (vesicular breath sound) is soft, low pitched, continuous, rustling in quality, more intense, and high pitched during inhalation than exhalation.

Stridor is a high-pitched sound that develops in the upper airway, mainly occurs when a person inhales, and can mostly be audible in the neck.

Rhonchi are low-pitched and continuous sounds similar to snoring sounds, occurring due to blockages in the lungs’ large airways.

Wheezing is a high-pitched, continuous sound resulting from blockages or when a person’s airways become constricted.

Fine and coarse crepitations are intermittent sounds normally audible during inhalation, sounding similar to bubbling, popping, or clicking noises. Fine crepitation occurs in the small airways, with soft tones and high pitch. It may occur more frequently during breathing than coarse crepitation and only during inhalation. Furthermore, coarse crepitation occurs in the larger bronchi tubes and is loud, low pitched, and lasts longer than fine crepitation; it mainly occurs during inhalation but can also occur during exhalation.

In summary, each breathing sound has a distinctive voice and tone found in different body positions. Including the other information from reviews, suggestions, and comments, all were used as strict information for the CHIM design and assembly and will be reported and described in the next section.

#### 2.1.3. Gagné’s Learning Theory

Gagné’s learning theory is widely recognized that it can be applied directly in the teaching and learning system by creating situations or events to create determination for learners [8,9], matching our aims.

Gagné’s learning theory includes nine steps: (1) gaining attention, (2) informing learners of the objective, (3) stimulating recall of prior learning, (4) presenting the stimulus, (5) providing learning guidance, (6) eliciting performance, (7) providing feedback, (8) assessing performance, and (9) enhancing retention and transfer. Therefore, it is excellent to be employed in this research. All nine steps were involved in the CHIM usage, instruction manual, and training in the laboratory.

#### 2.1.4. Hardware, Software, and Information Matching

Information from the experts and theory mentioned in Section 2.1.1, Section 2.1.2 and Section 2.1.3 was forwarded to expert engineers to match with the hardware, software, and program capabilities. They selected all readily available in the market and widely used to make the CHIM cheap and easy to maintain. Again, all will be presented and explained in the next section.

### 2.2. Design and Assembly

**Figure 3:**
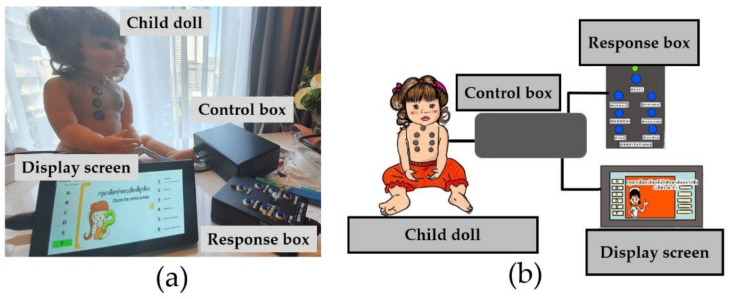
A CHIild Model (CHIM): (**a**) actual model and (**b**) diagram.

Using the information in Section 2.1, the CHIM was designed and assembled. Figure 3 shows the CHIM and supporting devices: (a) actual model and (b) diagram. Brief details explaining device functionality can be described as follows.

#### 2.2.1. Hardware

The hardware consists of a child doll, control box, response box, and display screen; wires and signal cables are connected all.

- Child doll

**Figure 4:**
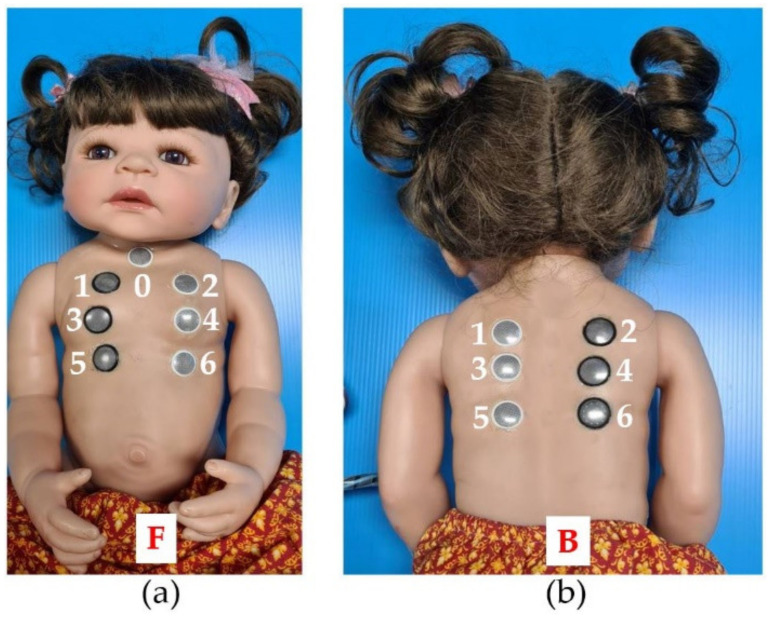
A child doll viewed on (**a**) the front and (**b**) the back.

A 60 × 23 cm^2^ size child doll with skin made of silicone vinyl was selected as a model. It was cute and shiny, so it attracted attention, achieving the 1st step of Gagné’s learning theory (gaining attention). The silicone is soft, flexible, and lifelike, better than other dolls made of plastic and fabric. This size can represent children under three months. Then, its chest and back were pierced to make thirteen holes at different positions: seven at the chest and six at the back. The piercing positions were assigned according to the advice of experts mentioned in Section 2.1.2. These positions clearly exhibit the breathing sounds frequently detected in most physical examinations of patients [10]. Therefore, a position assignment like this may help nursing students become familiar with the relationship between the position and breathing sounds and compensate for one of the SimBaby limitations, increasing their proficiency. Finally, all holes were inserted with earphones, which amplified the breathing sounds transmitted from the control box. Small gaps between the earphones and skin were filled with silicone glue to prevent leaked sounds and enhance durability. Figure 4 shows a complete child doll viewed on (a) the front and (b) the back. Moreover, numbers 0–6 and letters F and B were used to identify breathing sound types generated by the control box, explained later. F and B stand for the front and back sides, respectively, while numbers 0–6 represent positions. In actual use, the child doll had clothing for realism.

- Control box

**Figure 5:**
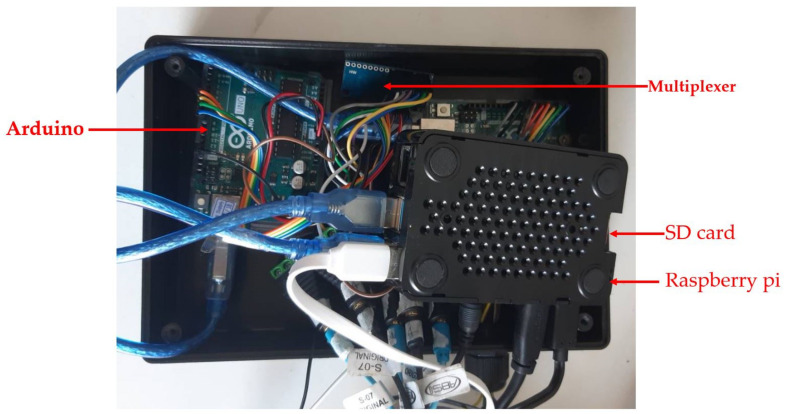
Sample devices inside control box.

All devices were controlled by the control box; therefore, it is an essential part of the CHIM containing Raspberry pi4, a multiplexer, Arduino, an SD card, an adapter, etc. Figure 5 shows the sample devices inside the control box.

**Figure 6:**
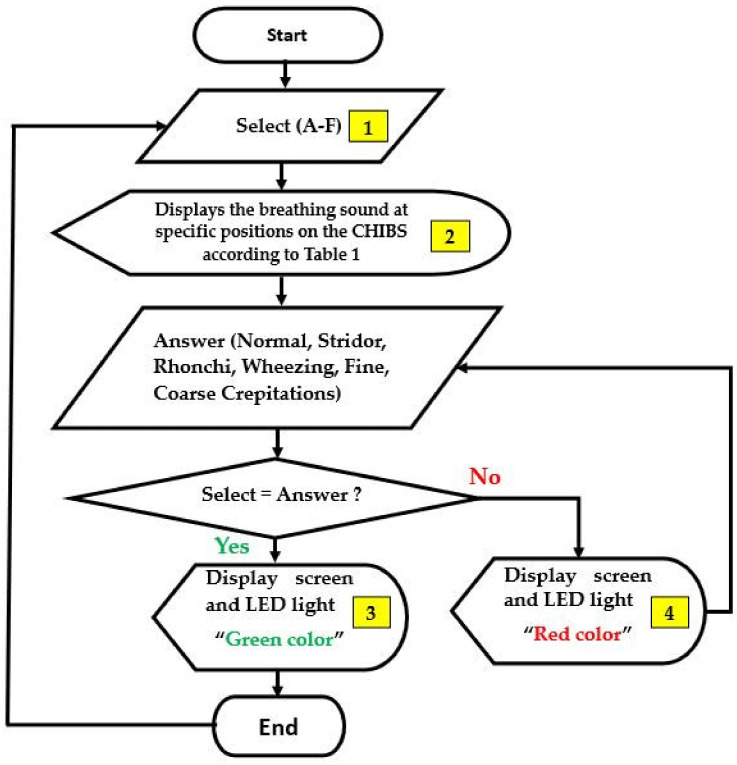
A flowchart describing the algorithm.

The Raspberry pi4 is a small computer working as input, output, and processing units. The algorithm that controlled the CHIM operations was written and coded in the C programming language, then embedded in the Raspberry pi4 operating system. The major operation accurately assigns the breathing sound types in Table 1 to match the earphones’ positions in Figure 4. Table 1 is the relationship between breathing sound types and positions mentioned in Section 2.1.2. In addition, Figure 6 shows a flowchart describing the algorithm. The small yellow boxes represent the display screen discussed later next.

The multiplexer or data selector is a device that selects digital inputs and forwards the selected input. In this research, it was employed to select the breathing sounds and transmit them to specific position outputs.

The Arduino is a microcontroller that works with a multiplexer to produce breathing sounds precisely as designed.

The SD card was used to store images, breathing sounds, codes, programs, etc., which were required to support the various device functions. Notably, the breathing sounds employed in Table 1 were representative sounds selected from more than 30 samples of online media by the experts mentioned in Section 2.1.2.

The adaptor was used for converting 220 V to 5 V AC.

In fact, the CHIM still has other devices, but they were not reported in this article since it is a prototype in the development process and upgrade for commercial products.

- Response box

**Figure 7:**
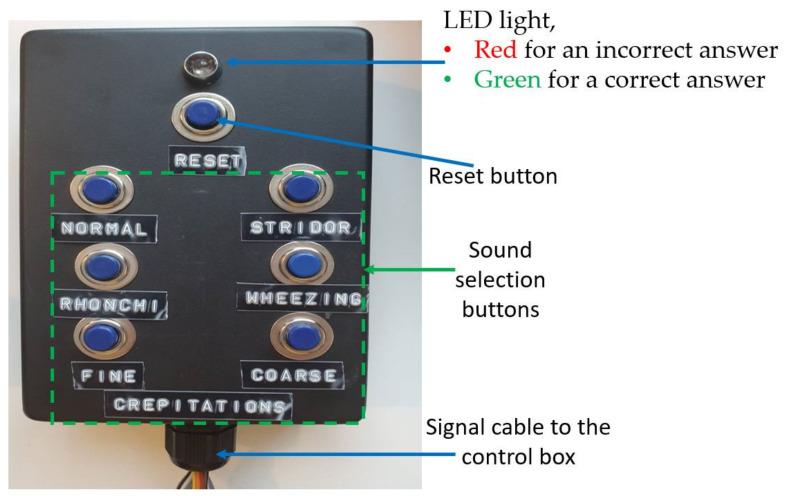
A response box.

The response box is for nursing students to enter their answers after using the stethoscope to listen to the breathing sounds on the CHIM in all positions. As seen in Figure 7, it has seven buttons and one LED light. Six buttons with the names of breathing sounds below are sound selection buttons to answer, feedback knowledge, and understanding from nursing students after auscultation. One button locates the top for a reset process. After nursing students choose their answers, the embedded programs in the control box will check the correctness and display the results on the display screen and LED light on the response box immediately. A green LED light for a correct answer and a red color for an incorrect answer. The control box functionality achieves the 7th step of Gagné’s learning theory (providing feedback).

- Display screen

**Figure 8:**
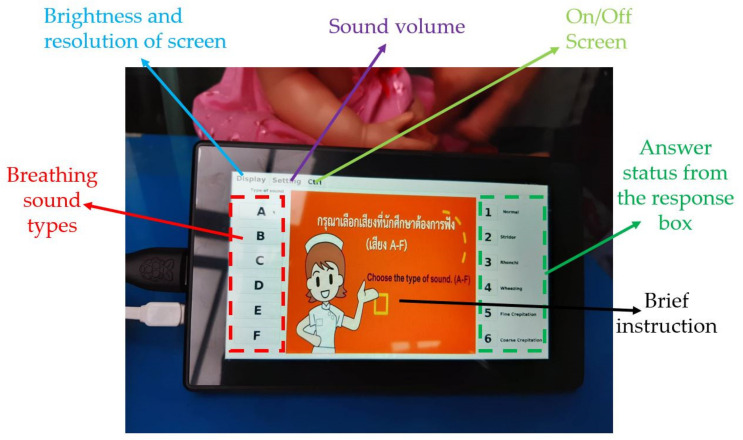
A display screen.

Every button press changes the display screen, which may make users confused. Therefore, QtCreator was employed to design the display screen to help the users’ ease of use. Figure 8 presents the display screen details. It includes many small boxes representing breathing sound type, answer status, display, setting, and control. For example, first, the breathing sound type boxes on the left allow nursing students to select the breathing sound types. Second, the answer status boxes on the right are the results of a user-selected button at the response box. Third, a display box on the top is for brightness and screen resolution settings. Fourth, the setting box is for a sound volume adjustment. Fifth, the control box is for turning on or off the CHIM. Finally, a brief instruction to guide users is in the middle of the screen, which depends on the user selection.

To use the CHIM, first, select the breathing sound on the left of a display screen. Then, place the stethoscope at various positions on the CHIM’s body and listen carefully. Next, choose the correct answer you think from the response box. After that, observe the color of the LED light on the box and the information on the display screen. It is green for correct and red for incorrect. If it is red, choose a new answer repeatedly until it is correct. Finally, you can select other breathing sound types to train yourself if it is correct or press the reset button. To understand the display screen, the small yellow boxes in Figure 6, and the response box operating, please download Display_Screen.pdf and Example.mp4 showing a usage example of both devices in Supplementary Materials.

#### 2.2.2. Software

In summary, there were three software employed in this research. First, the C programming language was used to write the algorithm for devices in the control box. Second, QtCreator was applied to create the display screen layout. The last, the Raspberry pi operating system, was used to connect precisely and control all devices working together.

All devices, including hardware and software, were assembled completely to be the CHIM. Then, the actual usage was tested until the authors were confident that it worked correctly and safely. The next step is to assess the reliability in terms of breathing sound types, positions, usage, and satisfaction by experts.

### 2.3. Verification and Assessment

The CHIM functionality was verified in the accuracy of breathing sound types, tones, voices, sound volumes, positions, and satisfaction by the medical doctor and the advanced practice nurse who have more experience in physical examination for a long time. They gave valuable comments and suggestions to improve the CHIM functionality in accordance with the anatomy and physiology. At the same time, an expert engineer in programming, embedded system, robot, and computing, checked the CHIM functionality in terms of the speed of user response, safety, error, and bugs in programming. His feedback was analyzed to enhance the CHIM’s stability, durability, and precision. As a result, the CHIM had many revisions and improvements until the three experts agreed to assess it.

Three experts assessed the CHIM quality and satisfaction of users. For the quality assessment, all breathing sounds were assessed on quality, volume, and occurring position. Since no quality assessment form has been found with similar objectives to this research, the authors assigned the contents and questions of this form. Therefore, the experts had to rate the quality in the assessment form as an integer from 1 to 4; 1 for irrelevant, 2 for somewhat relevant, 3 for quite relevant, and 4 for highly relevant. Finally, the quality assessment results were analyzed based on a content validity index (CVI) using the same method in [11].

Furthermore, the satisfaction assessment form was developed from Boonchoochuay’s work [12] to assess the satisfaction of using the CHIM, consisting of nineteen topics such as creative initiatives in further development, suitability with cost-effective use, suitability of materials used, safety, consistency with the lesson, and so on. The assessment score provided by the experts was assigned as an integer from 1 to 5; 1 for very dissatisfied, 2 for dissatisfied, 3 for neutral, 4 for satisfied, and 5 for very satisfied. The quality and satisfaction assessment results will be reported in Section 4.

For transparency, note that the three experts in this section differed from those mentioned in Section 2.1.

## 3. Skills and Satisfaction Examination Process

**Figure 9:**
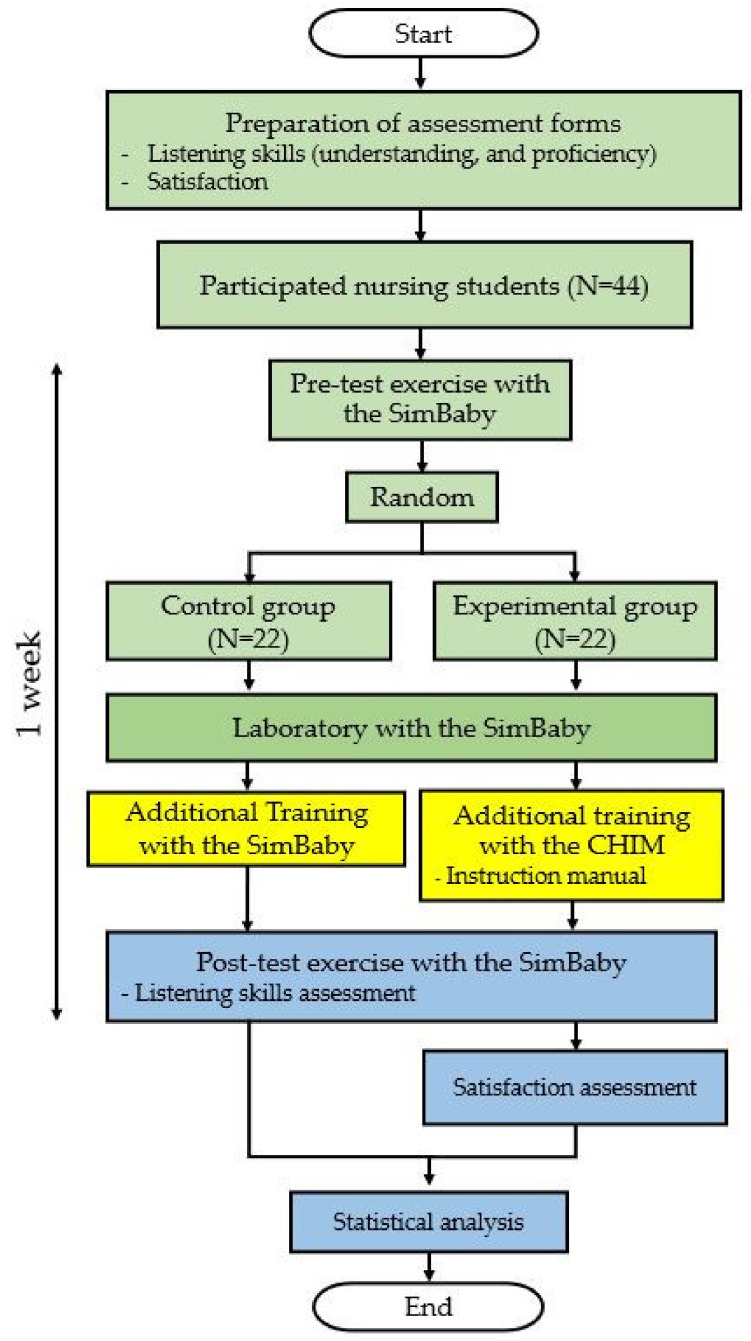
A flowchart of the skills and satisfaction examination process.

Figure 9 reports a flowchart for examining the effect of a CHIM on breath-sounds skills and satisfaction of nursing students consisting of pre-experiment, experiment, and post-experiment, presented in green, yellow, and blue colors, respectively, described below.

### 3.1. Pre-Experiment

In preparation for assessment forms to use in the examination, since nursing students have different backgrounds and experiences from the experts, the assessment forms were prepared to suit them. The pre-experiment consists of auscultation skills and satisfaction assessment forms. The auscultation skills assessment form was divided into two topics, understanding and proficiency. With a total score of 4, the understanding topic accessed the relationship between six breathing sounds and positions. With a total score of 6, the proficiency form assessed nursing students’ proficiency and skills, such as comparing left and right sounds, not listening through clothes, the closeness between the stethoscope and the CHIM’s skin, listening to both inhaling and exhaling sounds, and listening to all the positions. The authors proposed these assessment forms since no questionnaires from other similar work were found. Next, for satisfaction assessment, the experts’ form mentioned in 2.3 was adapted from the work of Nasaree [13] to match the nursing students with a total score of 5. 

This research is quasi-experimental, with two groups, pre-, and post-test designs, to study the CHIM outcomes on children’s auscultation skills and satisfaction. First, forty-four third-year junior nursing university students consented to participate. Then, they had a pre-test exercise using the SimBaby and assessed their auscultation skills of breathing sounds by listening and proficiency using the assessment form proposed by the authors mentioned above. The results were recorded, and the authors checked the accuracy. It was the first time they experienced the SimBaby. They used to listen to adults’ breathing sounds before but not children; therefore, they had real original understanding and proficiency without contamination in this step. Next, they were randomly assigned to control and experimental groups with the same number (N = 22). Finally, all had a laboratory learning respiratory system, clinical care, and especially, breathing sounds. Learning in the laboratory with the SimBaby is a regular course of study, even if without the CHIM. Table 2 reports basic information of participating nursing students before proceeding to the pre-test exercise with the SimBaby.

Table 2 implies that all nursing students had the same background and proficiency and had never been trained in listening to breathing sounds and auscultation skills before, which is suitable for this assessment.

### 3.2. Experiment

**Figure 10:**
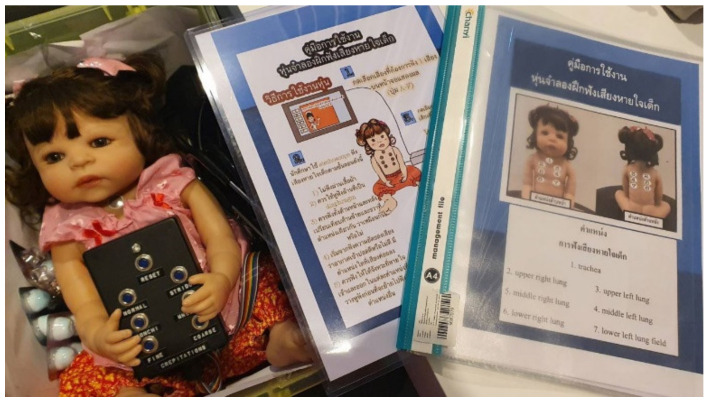
The CHIM and its instruction manual.

**Figure 11:**
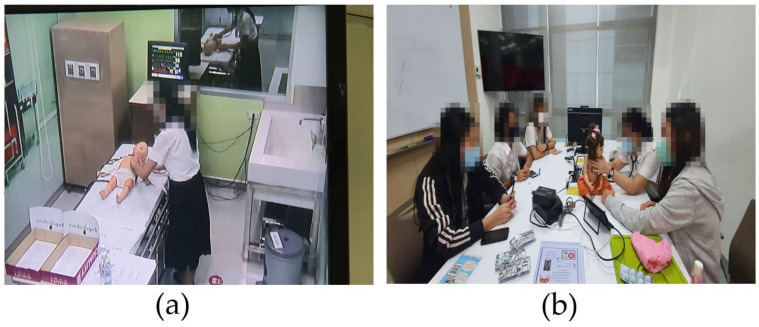
The atmospheres of additional training of (**a**) the control group with the SimBaby and (**b**) the experimental group with the CHIM.

The control group was allowed to perform additional training by themself using the SimBaby at their convenience. Similarly, the experimental group trained themselves with the CHIM simultaneously. They could retrain as needed. The retrain by themselves is consistent with the ninth step of Gagné’s learning theory. The preparation phase consists of three steps: gaining students’ attention, informing students of the objective, and recalling prior learning. The author introduced the CHIM, the objective of using it, and the benefits to students in gaining knowledge and experience of the auscultation skill. The instruction and practice phase is composed of four steps: presenting the stimulus, providing learning and guidance, eliciting performance, and providing feedback. The author provided students with instruction manuals on how to use the CHIM. A student can practice with the CHIM by choosing one of the breath sounds by pressing the A, B, C, D, E, or F buttons. The student can look at the manual to see if the answer was right or wrong. Students can also practice with a friend, who can then give feedback if the answer was right or wrong. The assessment and transfer phase is composed of two steps: assessing performance and enhancing retention and transfer. Finally, the students completed the post-test exercise with the SimBaby. Although the experiment did not contain any long-term practice data, the author hopes that students will continue using auscultation skills when practicing in clinics. In addition, Figure 10 shows the CHIM and its instruction manual for the experimental group. In addition, Figure 11 reveals the atmospheres of additional training of (a) the control group with the SimBaby and (b) the experimental group with the CHIM. Nursing students could train alone or in a group at their convenience. Significantly, the experimental group could retrain with the CHIM even in a classroom, co-working space, coffee shop, dormitory, canteen, etc., and not be afraid of damage since it was easy to fix. Every time and everywhere, learning with the CHIM is the highlight of the authors’ work intended.

### 3.3. Post-Test Experiment

After a week, both groups were assessed with the SimBaby using the same process as the pre-test experiment. Next, the auscultation skills were assessed and recorded. Then, only the experimental group assessed the satisfaction. Finally, the assessment results of auscultation skills were analyzed by the *t*-test statistics, while the descriptive statistics analyzed the assessment results of satisfaction. The assessment outcomes will be discussed in the next section. 

## 4. Results and Discussion

Assessment results were reported in two parts: the experts’ and nursing students’ assessments. For the nursing students, the assessments were processed based on two hypotheses: the first auscultation skill of the experimental group was higher than that of the control group after one week of the experiment, and the second auscultation skill of the post-test was higher than the pre-test in the experimental group.

### 4.1. Experts’ Assessment

Using the assessment form mentioned in Section 2.3 to evaluate the CHIM quality, the sound types were accurate, sound loudness levels were appropriate, and the sound positions were correct. The assessment score results from three experts were averaged, calculated, and analyzed to the content validity index (CVI), the same method presented in [11]. In the analysis, the calculated CVI was 0.94, higher than 0.8. Note that the maximum CVI is 1, so the calculated CVI was interpreted as good content accuracy and the CHIM quality using the same criteria as [14].

Moreover, the average satisfaction score evaluated from 19 topics was 4.39, implying that the experts were very satisfied with the highest level, using the CHIM. The detail of the nineteen topics is close to that of nursing students reporting in the next section. In the detailed analysis, the maximum score of 5 (perfect score) was used three topics: creative initiatives in further development, appropriate and cost-effective use, and safety. At the same time, the minimum score of 3.6 was rated on two topics: suitability of materials used and completeness of the anatomy contents in the instruction manual. Other topics were rated between the maximum and minimum scores but were not reported in detail because they did not affect our conclusion in this section.

From the above, the quality and satisfaction assessment results concluded that the CHIM could practically be employed in the training skills of nursing students, as expected.

### 4.2. Nursing Students’ Assessment

This section is divided into two parts: auscultation skills and satisfaction assessments.

#### 4.2.1. Auscultation Skills

Table 3 reports both groups’ pre- and post-test results, with a total score of 10. The control group’s pre-test score was 5.39, with an S.D. of 1.12 and a range between 3.33 and 7.67. The post-test score was 5.27, with an S.D. of 1.25 and a range between 3.00 and 8.00. Moreover, the experimental group’s pre-test score was 4.95, with an S.D. of 1.20 and a range between 2.67–7.00. The post-test score was 6.84, with an S.D. of 1.25 and a range between 3.33 and 9.00. Next, the statistical analysis results will be discussed with two hypotheses.

Using Table 3 for input calculation, Table 4 reports the independent *t*-test statistical results in a post-test experiment of both groups for testing the first hypothesis.

The first hypothesis is that the auscultation skill of the experimental group was higher than that of the control group after one week of the experiment. In Table 4, the mean value in the experimental group was 6.84, with an S.D. of 1.64, while it was 5.27 in the control group, with an S.D. of 1.25. Both were statistically significant differences, with a *p*-value of 0.001. Since the *p*-value was lower than 0.5 (*p* < 0.05), this assessment accepted the first hypothesis.

Similarly, Table 5 reports the independent *t*-test statistical results in the experimental group’s pre- and post-test experiments to test the second hypothesis.

The 2nd hypothesis is that the auscultation skill of the post-test was higher than the pre-test in the experimental group. From Table 5, the mean value in the post-test was 6.848, with an S.D. of 1.648, while it was 4.955, with an S.D. of 1.200 in the pre-test. Both were statistically significant differences, with a *p*-value of 0.000. Therefore, according to *p* < 0.05, this assessment accepted the 2nd hypothesis. The acceptance of both hypotheses can be discussed to link with the other work reported below.

The auscultation skills of breathing sounds need to be retrained and repeated to achieve proficiency, consistent with two previously reported research findings. First, Bernadi et al. [15] reported that after fourth-year medical students listened to breathing sounds for 1 h, the comparison between the assessment results before and after the experiment showed no statistically significant difference at the 0.05 level. Therefore, it may imply that the longer the auscultation skills, the higher the proficiency. Second, Thorndike [16] stated that when learners are physically and mentally ready, retraining and repeating with understanding will make learning stable and sustainable.

Next, using the stethoscope for listening and the response box for replying is a two-way interaction, giving more confidence and enhancing learning speed. This discussion is consistent with the outcomes reported by Boonchoochuay [12] and PunYoo [17]. For example, an innovative model for practicing phlegm suction skills, entitled the RTAFNC Suction Model, can interact with the users through an LED light. A blinking light indicates a correct answer but does not change for an incorrect answer [12]. Another point is that the use of pediatric computer-aided instruction on medication skills with question-and-answer feedback significantly improved nursing students’ skills [17].

Finally, applying Gagné’s learning theory to develop a novel method for training enhances the target learning skills. It is supported by the work of Wu et al. [18], who developed a virtual reality (VR) game. They used Gagne’s learning model to improve universal precaution for needlestick or sharp injury prevention and decrease the rates of needle sticks or sharps injuries in new-coming medical and nursing interns in Taiwan. Similarly, the CHIM is a new way similar to the VR games in [18]. The CHIM is a stimulating tool for nursing students’ independent learning. Since the CHIM will be duplicated to distribute them sufficiently to nursing students, the authors hope that continuing with their self-practice after the experiment will improve their auscultation skills for taking care of real patients when they turn into the clinical practice on the ward.

#### 4.2.2. Satisfaction

With a total score of 5 on each topic, Table 6 reports satisfaction assessment topics and the descriptive statistical results in the experimental group. They were rated as very satisfied in seven out of ten topics. For example, the maximum mean value was 4.86 in a topic that the CHIM gives nursing students the freedom of learning. On the other hand, the minimum mean value was 4.18 in a topic where the breathing sounds are clear and easy to listen to, consistent with one of the experts’ suggestions. Therefore, nursing students were very satisfied (the highest satisfied level) with the CHIM.

Finally, keep in mind that the CHIM was designed to be capable, as suggested by experts from many fields, of long-term experience in children’s auscultation and has only breathing sounds capability. Thus, it certainly cannot replace a realistic state of patients or equivalent to the SimBaby. Therefore, additional research into healthcare and engineering, together with updated technology, will help advance the CHIM’s capabilities, which is a further goal of the authors.

## 5. Conclusions, Suggestions, and Limitations

Auscultation is a necessary procedure in evaluating children’s respiratory diseases. Therefore, nursing students should be trained on breathing sounds and learn about the respiratory system before practicing in a patient unit. Usually, nursing students in Thailand’s leading universities are trained in auscultation skills, listening to breathing sounds using the SimBaby. However, due to the lack of the number of SimBaby mannequins, the CHIld Model (CHIM), an innovation emulating breathing sounds, was invented and researched based on children’s anatomy, physiology, and Gagne’s learning theory. As a collaboration between engineering and nursing fields, the research was divided into the CHIM invention process and the nursing students’ skills and satisfaction examination process. First, after completing the CHIM invention, expert assessment results revealed satisfaction with the use. In addition, they confirmed that it was reliable with good quality to be employed in the training skills of nursing students. Second, after implementing the CHIM in training skills for nursing students, the assessment results of auscultation skills and satisfaction showed that the auscultation skills and proficiency were significantly enhanced in statistics. Additionally, usage satisfaction was achieved at the highest level with statistical significance. Therefore, the CHIM was suitable to be actually used to support the learning of the respiratory system and solve the lack of the SimBaby, as intended.

However, a valuable suggestion from the experts and nursing students coherently stated that the CHIM’s skin was stiffer and less flexible than that of the SimBaby, resulting in the difficulty of the stethoscope to adhere closely to the CHIM’s skin since the adhesion would make it easier to hear the breathing sounds. An additional suggestion indicated that the breathing sound types and sound-generating positions were unclear and should be added more. Therefore, after analyzing the suggestions, the first suggestion changed the type of skin materials closer to the SimBaby, while in the latter, reengineering and redesign were the solutions. All will be applied as information for further improvement. Since the prototyping budget was approximately 0.004% compared to the buying price of the SimBaby, which is low cost, the CHIM is expected to be duplicated and implemented for actual use and commercial purposes soon.

Nevertheless, some limitations and flaws in the experiment decrease the credibility of assessment results in Table 3, Table 4, Table 5 and Table 6, such as having only one week for the experiment and the laboratory procedure in usage. Since the time allowed for this experimental program is short, at only one week, based on the practicum schedule for nursing students, they need to rotate to practice in a patient’s unit quickly. Therefore, the expansion of experimental time may affect their practicum schedule. Moreover, the opening of the laboratory is from 8.00 a.m. to 8.00 p.m., and under the staff’s observation, there are strict regulations. The mentioned strict regulations were a standard to ensure students’ safety and avoid possible damage to the SimBaby and other laboratory instruments that the authors cannot violate. Absolutely, suppose the mentioned limitations are eliminated, such as extending the experimental time and opening the laboratory for 24 h without the staff for the control group. In that case, the credibility of assessment results will be increased. However, the assessment results initially indicated that the CHIM could assist nursing students in learning with satisfaction, as the authors intended.

## Figures and Tables

**Table 1:** The relationship between breathing sound types and positions from experts’ advice.

Breathing Sound Type	Position in Figure 4
1. Normal	6F
2. Stridor	1F
3. Rhonchi	3F
4. Wheezing	5B, 6B
5. Fine crepitation	3B, 4B
6. Coarse crepitation	3F

**Table 2:** Basic information of participating nursing students (N = 44).

Personal Characteristic	Control Group (N = 22)		Experimental Group (N = 22)	
	Number	%	Number	%
Sex				
Male	0	0	1	4.55
Female	22	100	21	95.45
Age				
20	7	31.81	12	54.55
21	13	59.09	8	36.36
22	1	4.55	2	9.09
25	1	4.55	0	0
Mean (SD)	20.91 (1.06)		20.55 (0.67)	

**Table 3:** The auscultation skill assessment results in control (Con.) and experimental (Exp.) groups.

	Pre-Test Experiment				Post-Test Experiment			
	Con.		Exp.		Con.		Exp.	
	Mean	S.D.	Mean	S.D.	Mean	S.D.	Mean	S.D.
Score	5.39	1.12	4.95	1.20	5.27	1.25	6.84	1.64
Range	(3.33–7.67)		(2.67–7.00)		(3.00–8.00)		(3.33–9.00)	

**Table 4:** The Independent *t*-test statistical results in a post-test experiment of both groups.

Group	N	Auscultation Skills for the Post-Test Experiment		*t*	*p*-Value
		Mean	S.D.		
Con.	22	5.27	1.25		
Exp.	22	6.84	1.64	−3.568	0.001 *

* *p* < 0.05.

**Table 5:** The Independent *t*-test statistical results in pre- and post-tests of the experimental group.

Auscultation Skill	Mean	S.D.	*t*	*p*-Value
Pre-test	4.955	1.200		
Post-test	6.848	1.648	−4.792	0.000 *

* *p* < 0.05.

**Table 6:** The satisfaction assessment topics and results in the experimental group.

Satisfaction Topics on the CHIM:	(Range)	Mean	S.D.	Level
1. Gives you the freedom of learning.	(4–5)	4.86 *	0.351	Very satisfied
2. Help you learn fun.	(4–5)	4.68	0.477	Very satisfied
3. Help you learn yourself.	(4–5)	4.64	0.492	Very satisfied
4. Motivate you to learn.	(3–5)	4.64	0.581	Very satisfied
5. Help you to learn quickly and easily.	(3–5)	4.55	0.596	Very satisfied
6. Is an exciting way of learning.	(3–5)	4.55	0.671	Very satisfied
7. Support you with a positive attitude toward learning.	(4–5)	4.50	0.512	Very satisfied
8. Help you to train each step of auscultation skills properly.	(4–5)	4.45	0.510	Satisfied
9. Help you to understand the lesson contents better.	(3–5)	4.36	0.581	Satisfied
10. Breathing sounds are clear and easy to listen to.	(3–5)	4.18	0.664	Satisfied

* The maximum score.

## Data Availability

Not applicable.

## Supplementary Materials

The following supporting information can be downloaded at: https://www.mdpi.com/article/10.3390/healthcare10071165/s1, Display_Screen.pdf, and Example.mp4.
