# Supplementary material for: The Effect of a Child Model on Breath-Sounds Examination Skills and Satisfaction on Nursing Students
Source: Healthcare (Basel). 2022 Jun 22;10(7):1165. doi: 10.3390/healthcare10071165 (PMC9323406; doi:10.3390/healthcare10071165)
Supplement: Supplementary file 1 [file healthcare-10-01165-s001.zip › Display_Screen.pdf]

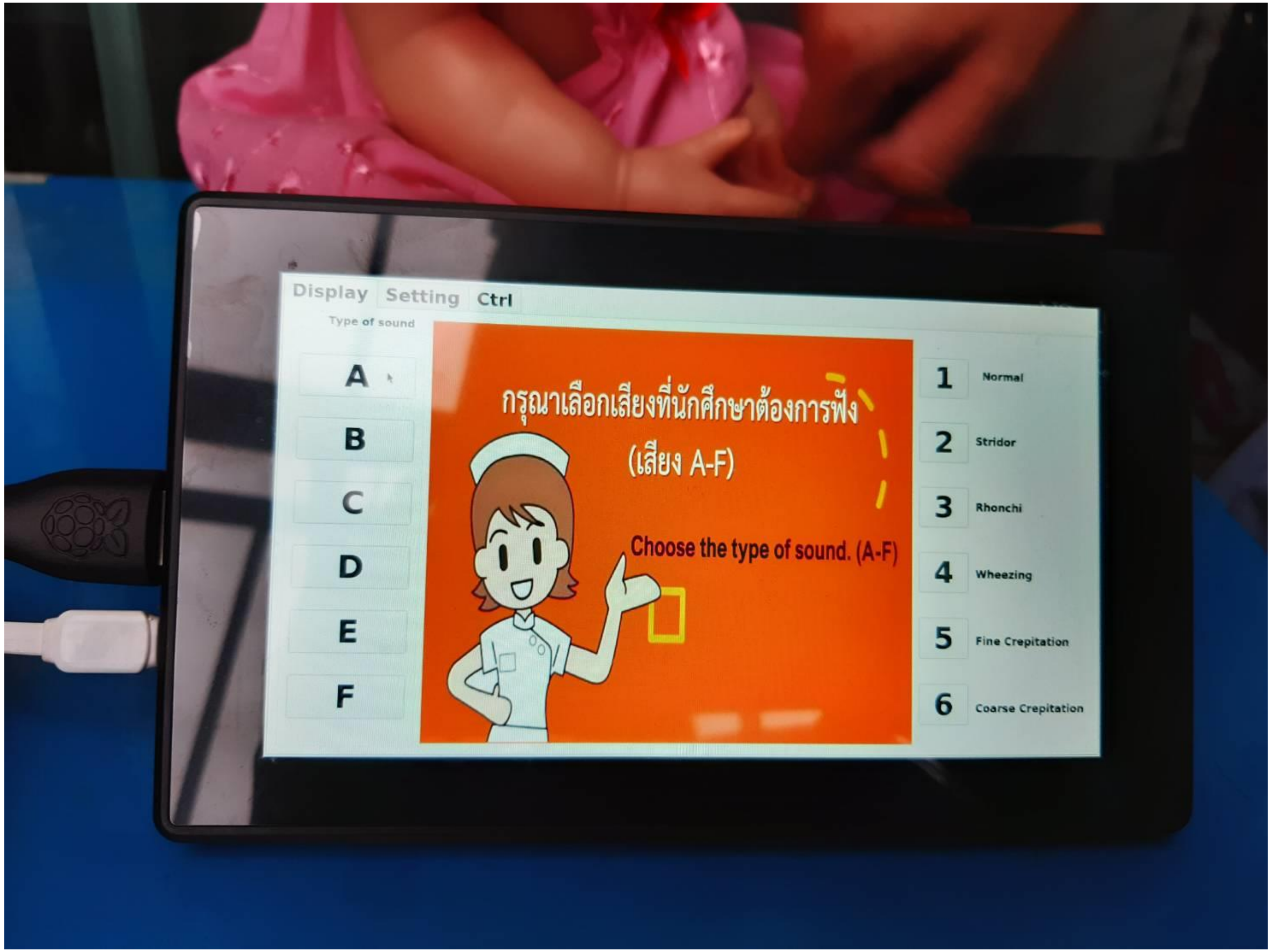

1

Tap on the screen button A – F to select a breathing sound.

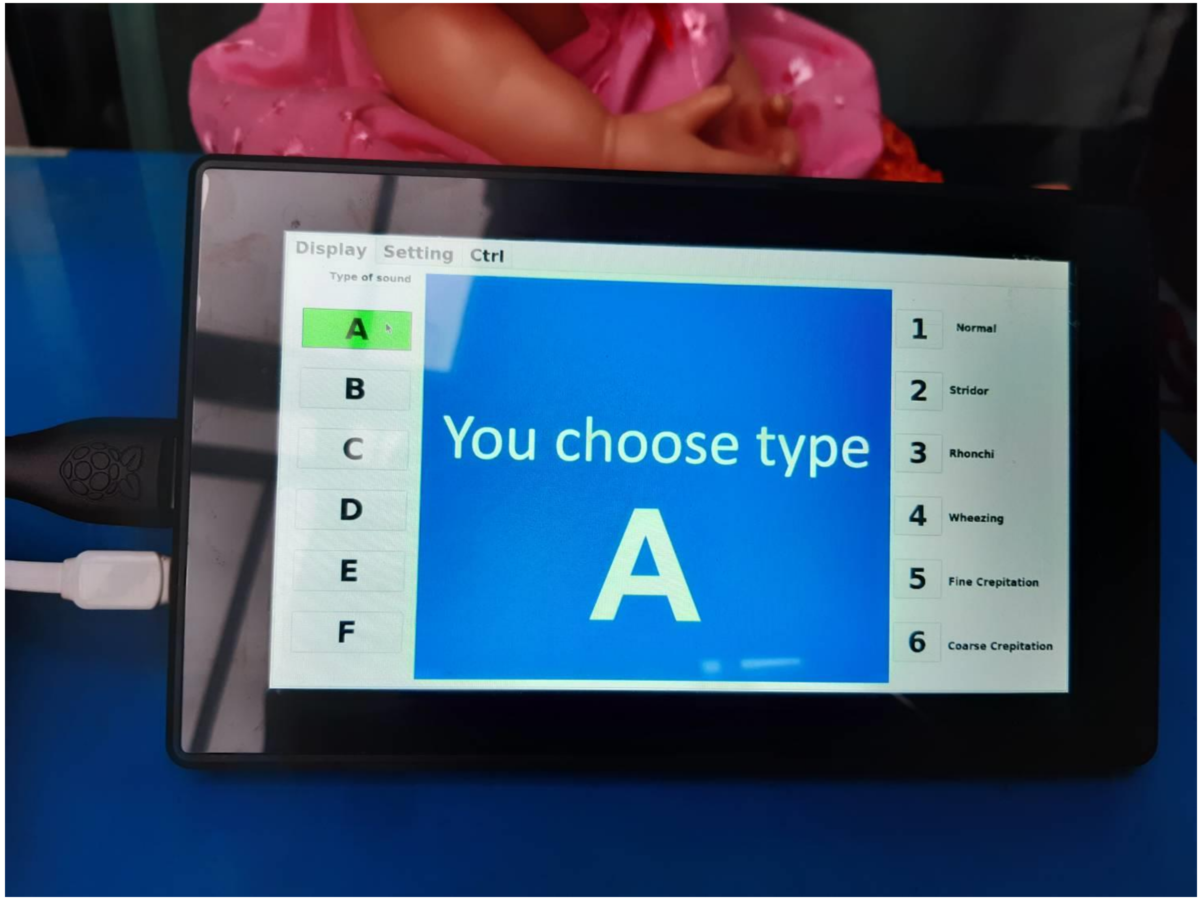

2

For example, students choose a type A breathing sound (Normal lung sound), so the selection result displays on this screen. Immediately, the Normal lung sound is generated at a position of 6F. Nursing students have to use a stethoscope to examine the sound at every position on the CHIBS and then use their knowledge gained from the laboratory to decide which sound it is. In addition, the small boxes on the left shows the confirmation of choosing A .

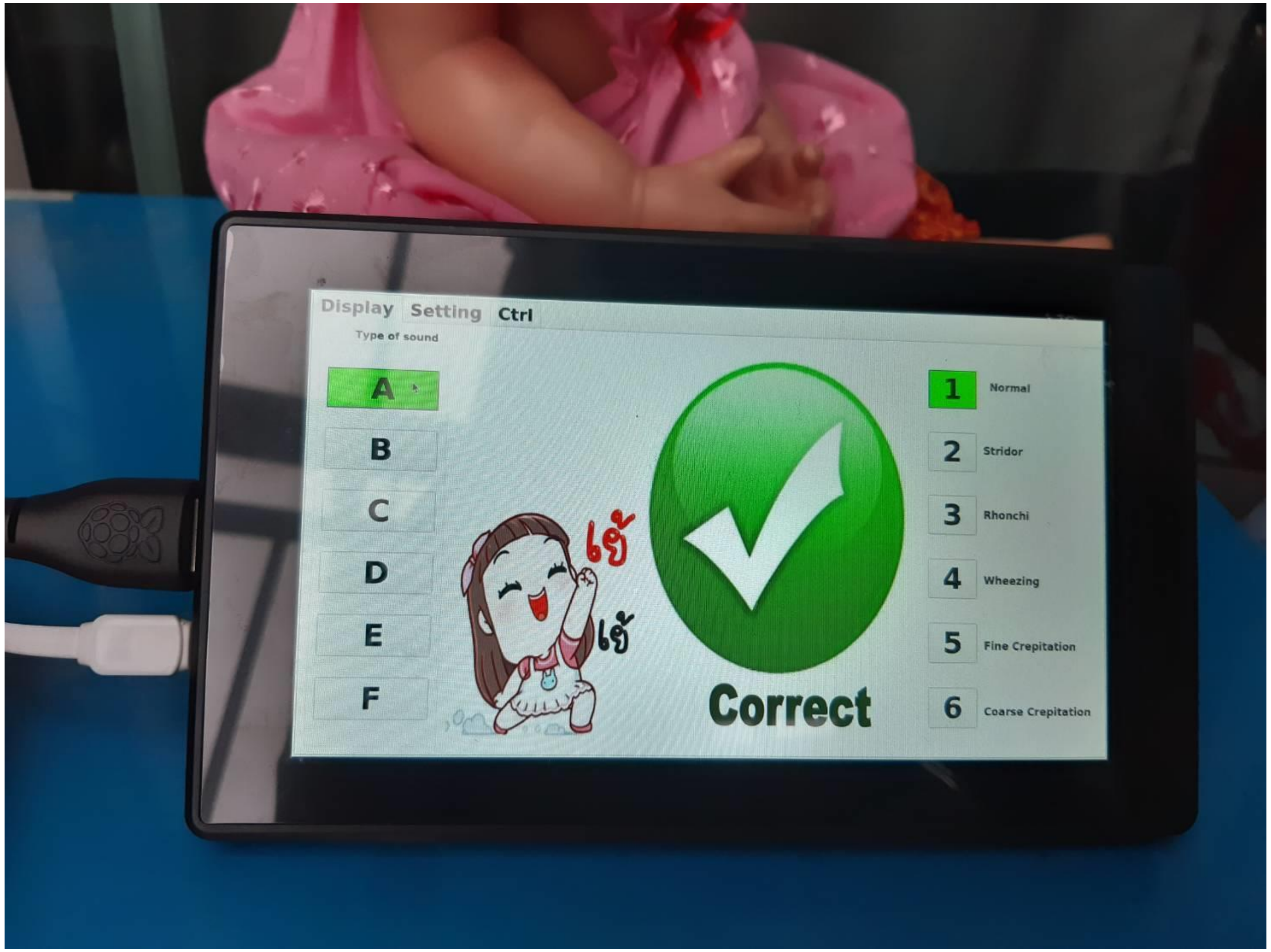

3

Once students are confident in their answers, they must select an answer in the response box. Small boxes on the right show the students' answer from the response box. This screen displays results for a correct answer.

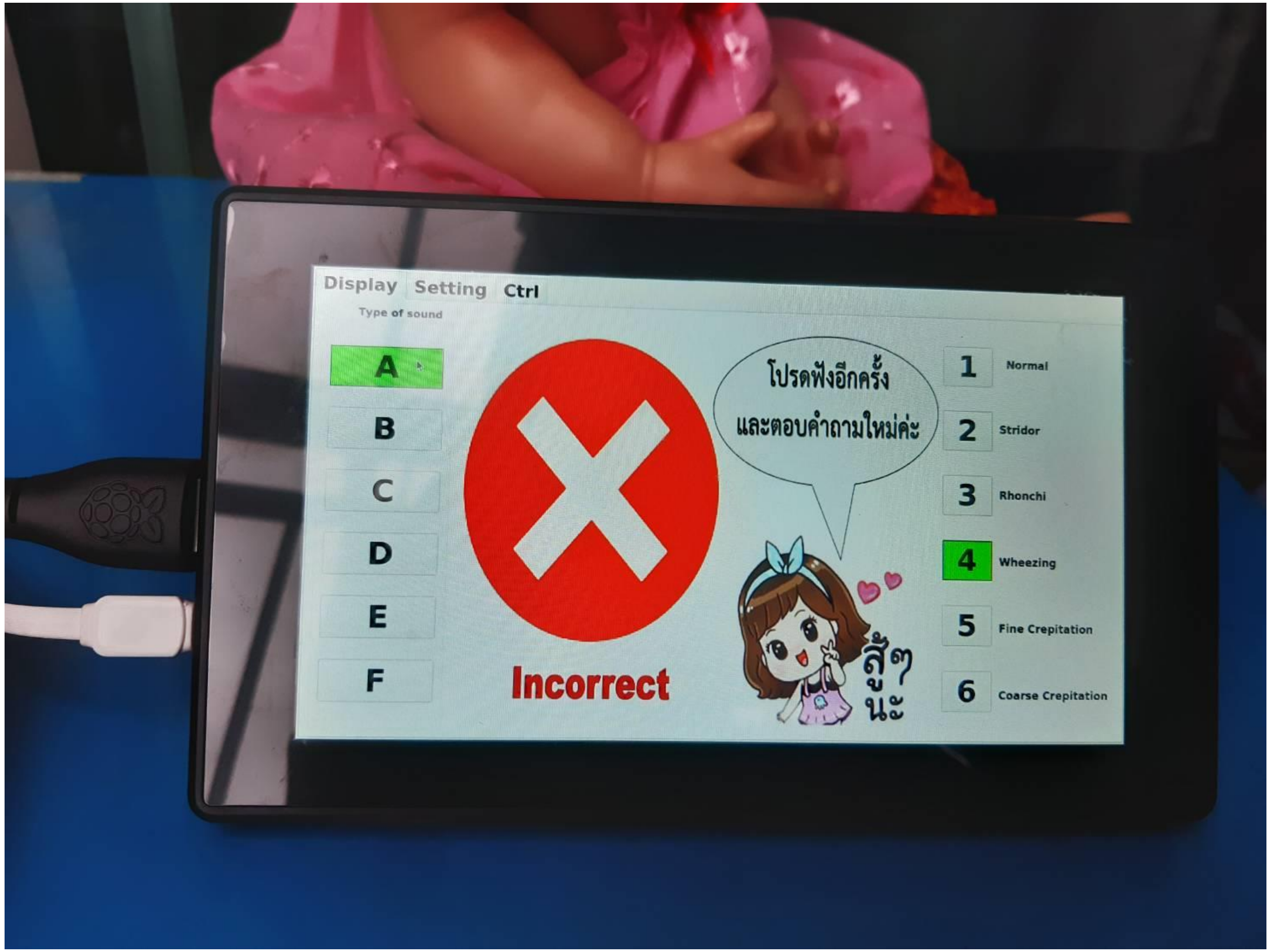

4

This is the screen for an incorrect answer because the students choose 4 (Wheezing) instead of 1 (Normal). This screen remains until the student selects the correct answer.

Then press a Reset button on the response box to repeat the process and return to **1** page for choosing other sounds.
